# Supplementary material for: Exploring Sustainable Development Pathways for Agri-Food Supply Chains Empowered by Cross-Border E-Commerce Platforms: A Hybrid Grounded Theory and DEMATEL-ISM-MICMAC Approach
Source: Foods. 2023 Oct 26;12(21):3916. doi: 10.3390/foods12213916 (PMC10647628; doi:10.3390/foods12213916)
Supplement: Supplementary file 1 [file foods-12-03916-s001.zip › foods-2635053-supplementary.pdf]

Supplementary Material

Table S1. Direct Influence Matrix Z

NO	S1	S2	S3	S4	S5	S6	S7	S8	S9	S10	S11	S12	S13	S14	S15	S16	S17	S18
S1	0	3.7	3.3	2.4	2.4	2.6	3.2	2.8	2.4	2.5	2.2	2.5	2.4	3.3	2.3	2.1	3	2.4
S2	2.4	0	3.1	2.4	2.8	1.7	2.5	2.5	2.5	2.3	2	2.3	1.9	2.4	2.5	2.4	2.1	2.2
S3	2.1	3	0	2.2	2.7	2.1	2.2	2.3	2.5	2.1	1.3	2	2.1	2.2	2.3	1.6	2.4	2
S4	1.7	2.9	3.1	0	2.5	2.5	2	2.1	2.2	2.1	1.7	2.5	2.2	2	1.8	2.2	2.2	2.2
S5	1.9	3.4	2.8	2.6	0	2.8	2.9	2.2	2.2	2.7	3	2.8	3.3	2.1	2.5	3.2	2.9	2.8
S6	1.6	2	2.6	2.5	3	0	2.4	2.1	1.7	1.9	2.2	2.2	2.7	2.5	2.4	1.9	2.1	2.1
S7	2.2	2.6	2.6	1.9	1.7	2.2	0	2.8	2.8	2	1.8	2	1.9	1.9	2.2	1.2	2	2.1
S8	2.1	2.9	2.7	1.7	1.7	1.6	2.5	0	3.3	1.8	2	2.8	2.7	1.8	1.8	1.2	2.1	2.3
S9	1.6	2.6	2.3	1.5	1.2	1.6	2.1	2.9	0	1.6	2	1.9	3.2	1.7	1.8	1.8	2.4	1.9
S10	2.2	2.9	2.7	1.8	1.9	2	2.2	2.4	2.4	0	1.5	3.1	2	1.6	2.3	1.7	1.7	2.2
S11	1.8	3.3	2.8	1.4	2.6	2.5	2.4	2.2	2.7	2.2	0	2.2	3.6	2.3	1.4	1.8	2.6	2.7
S12	1.7	3	2.5	2.1	2.3	2	2.7	2.5	2.2	2.7	3	0	2.5	2	2.2	1.5	1.9	2.4
S13	2	2.4	2.2	1.6	1.9	2.2	2.5	2.4	3.3	2.1	2.7	2.3	0	2.2	1.9	1.7	2.2	2.4
S14	2.3	2.8	2.5	2.1	1.9	1.9	2.4	2.2	2.3	1.7	2.4	1.9	2.3	0	2.4	2.1	2.7	3.2
S15	1.8	3.4	2.8	2	2.3	2.4	2	1.8	2.2	3.1	1.7	2.1	2.5	2.5	0	1.7	2.4	1.7
S16	2	3	2.4	3	2.8	2.5	1.8	1.7	2.5	1.8	2	1.7	1.9	2	1.9	0	2.3	2.8
S17	2.3	3.2	2.8	1.8	2.2	2.1	1.7	2.5	2.9	2.2	2.2	1.9	2.7	2.7	2.1	1.9	0	2.1
S18	2.2	2.2	2	2	2.1	2.6	2.3	2.2	2.6	2.4	2.2	1.8	2.4	2	1.8	2.5	2.9	0

Table S2. Comprehensive Influence Matrix T

NO	S1	S2	S3	S4	S5	S6	S7	S8	S9	S10	S11	S12	S13	S14	S15	S16	S17	S18
S1	0.256	0.437	0.402	0.311	0.333	0.330	0.363	0.355	0.369	0.329	0.314	0.335	0.363	0.345	0.316	0.288	0.358	0.343
S2	0.276	0.322	0.360	0.282	0.309	0.281	0.315	0.315	0.334	0.294	0.280	0.299	0.318	0.296	0.289	0.266	0.307	0.306
S3	0.255	0.360	0.277	0.262	0.289	0.272	0.291	0.293	0.315	0.273	0.250	0.276	0.303	0.275	0.269	0.236	0.295	0.284
S4	0.251	0.364	0.345	0.220	0.290	0.284	0.291	0.293	0.313	0.277	0.262	0.290	0.309	0.275	0.263	0.251	0.295	0.292
S5	0.299	0.436	0.396	0.319	0.288	0.338	0.360	0.346	0.369	0.337	0.333	0.345	0.385	0.325	0.323	0.313	0.360	0.355
S6	0.249	0.347	0.335	0.272	0.300	0.234	0.300	0.294	0.304	0.274	0.272	0.285	0.320	0.286	0.275	0.246	0.294	0.291
S7	0.249	0.342	0.320	0.248	0.261	0.265	0.237	0.294	0.311	0.263	0.251	0.268	0.290	0.261	0.259	0.220	0.278	0.277
S8	0.253	0.356	0.329	0.249	0.267	0.260	0.295	0.244	0.329	0.265	0.262	0.290	0.313	0.265	0.257	0.225	0.287	0.287
S9	0.227	0.327	0.301	0.229	0.240	0.243	0.268	0.284	0.242	0.244	0.245	0.254	0.303	0.246	0.240	0.222	0.274	0.261
S10	0.253	0.354	0.327	0.250	0.270	0.266	0.287	0.291	0.308	0.226	0.250	0.294	0.296	0.259	0.265	0.234	0.277	0.284
S11	0.267	0.391	0.357	0.264	0.307	0.299	0.316	0.312	0.342	0.294	0.241	0.300	0.355	0.296	0.270	0.257	0.319	0.318
S12	0.258	0.375	0.342	0.270	0.293	0.282	0.313	0.309	0.322	0.297	0.294	0.247	0.324	0.282	0.278	0.244	0.297	0.304
S13	0.256	0.354	0.327	0.253	0.277	0.278	0.301	0.300	0.335	0.277	0.281	0.286	0.265	0.279	0.264	0.241	0.295	0.296
S14	0.269	0.371	0.341	0.270	0.285	0.280	0.307	0.303	0.324	0.277	0.282	0.285	0.320	0.241	0.281	0.256	0.313	0.319
S15	0.256	0.377	0.342	0.264	0.289	0.285	0.294	0.291	0.317	0.300	0.264	0.286	0.318	0.288	0.229	0.244	0.302	0.285
S16	0.258	0.368	0.333	0.284	0.298	0.287	0.289	0.287	0.321	0.273	0.269	0.276	0.306	0.277	0.267	0.208	0.300	0.306
S17	0.270	0.381	0.349	0.265	0.292	0.284	0.295	0.310	0.337	0.287	0.279	0.287	0.329	0.297	0.277	0.253	0.259	0.299
S18	0.262	0.352	0.325	0.263	0.283	0.288	0.299	0.297	0.324	0.284	0.273	0.278	0.316	0.277	0.264	0.258	0.311	0.248

Table S3. Reachability Matrix M

NO	S1	S2	S3	S4	S5	S6	S7	S8	S9	S10	S11	S12	S13	S14	S15	S16	S17	S18
S1	1	1	1	1	1	1	1	1	1	1	1	1	1	1	1	0	1	1
S2	0	1	1	0	1	0	1	1	1	1	0	1	1	1	0	0	1	1
S3	0	1	1	0	0	0	0	0	1	0	0	0	1	0	0	0	1	0
S4	0	1	1	1	0	0	0	0	1	0	0	0	1	0	0	0	1	0
S5	1	1	1	1	1	1	1	1	1	1	1	1	1	1	1	1	1	1
S6	0	1	1	0	1	1	1	1	1	0	0	0	1	0	0	0	1	0
S7	0	1	1	0	0	0	1	1	1	0	0	0	0	0	0	0	0	0
S8	0	1	1	0	0	0	1	1	1	0	0	0	1	0	0	0	0	0
S9	0	1	1	0	0	0	0	0	1	0	0	0	1	0	0	0	0	0
S10	0	1	1	0	0	0	0	0	1	1	0	1	1	0	0	0	0	0
S11	0	1	1	0	1	1	1	1	1	1	1	1	1	1	0	0	1	1
S12	0	1	1	0	0	0	1	1	1	1	1	1	1	0	0	0	1	1
S13	0	1	1	0	0	0	1	1	1	0	0	0	1	0	0	0	1	1
S14	0	1	1	0	0	0	1	1	1	0	0	0	1	1	0	0	1	1
S15	0	1	1	0	0	0	1	0	1	1	0	0	1	0	1	0	1	0
S16	0	1	1	0	1	0	0	0	1	0	0	0	1	0	0	1	1	1
S17	0	1	1	0	0	0	1	1	1	0	0	0	1	1	0	0	1	1
S18	0	1	1	0	0	0	1	1	1	0	0	0	1	0	0	0	1	1

Table S4. Intersection of Reachable Sets and Preceding Sets

NO	Reachable Set(R)	Advance Set(Q)	Intersection Set(A=R∩Q)
S1	1,2,3,4,5,6,7,8,9,10,11,12,13,14,15, 17,18	1,5	1,5
S2	2,3,5,7,8,9,10,12,13,14,17,18	1,2,3,4,5,6,7,8,9,10,11,12,13,14,15,16 ,17,18	2,3,5,7,8,9,10,12,13,14,1 7,18
S3	2,3,9,13,17	1,2,3,4,5,6,7,8,9,10,11,12,13,14,15,16 ,17,18	2,3,9,13,17
S4	2,3,4,9,13,17	1,4,5	4
S5	1,2,3,4,5,6,7,8,9,10,11,12,13,14,15, 16,17,18	1,2,5,6,11,16	1,2,5,6,11,16
S6	2,3,5,6,7,8,9,13,17	1,5,6,11	5,6
S7	2,3,7,8,9	1,2,5,6,7,8,11,12,13,14,15,17,18	8,2,7
S8	2,3,7,8,9,13	1,2,5,6,7,8,11,12,13,14,17,18	8,2,13,7
S9	2,3,9,13	1,2,3,4,5,6,7,8,9,10,11,12,13,14,15,16 ,17,18	9,2,3,13
S10	2,3,9,10,12,13	1,2,5,10,11,12,15	2,10,12
S11	2,3,5,6,7,8,9,10,11,12,13,14, 17,18	1,5,11,12	11,12,5
S12	2,3,7,8,9,10,11,12,13,17,18	1,2,5,10,11,12	11,2,10,12
S13	2,3,7,8,9,13,17,18	1,2,3,4,5,6,8,9,10,11,12,13,14,15,16,1 7,18	2,3,8,9,13,17,18
S14	2,3,7,8,9,13,14,17,18	1,2,5,11,14,17	17,2,14
